# GLP-1, GIP, and Glucagon Receptor Agonism at the Obesity–Neurodegeneration Interface: A Narrative Review

**DOI:** 10.3390/ijms27198738

**Published:** 2026-09-30

**Authors:** Elena Popa, Andrei Emilian Popa, Vladimir Poroch, Thomas Gabriel Schreiner, Ana Maria Slanina, Mihaela Poroch, Alexandru Matei Hatneanu, Monica Iuliana Ungureanu, Antoneta Dacia Petroaie, Agnes Iacinta Bacusca, Elena Adorata Coman

**Affiliations:** 1 Faculty of Medicine, “Grigore T. Popa” University of Medicine and Pharmacy, 16 Universitatii Str., 700115 Iasi, Romania; elena.popa@umfiasi.ro (E.P.); antoneta.petroaie@umfiasi.ro (A.D.P.);; 2 Department of Preventive Medicine and Interdisciplinarity, “Grigore T. Popa” University of Medicine and Pharmacy, Universitatii Str. 16, 700115 Iasi, Romania; 3 “Prof. Dr. Nicolae Oblu” Emergency Clinic Hospital, 700309 Iasi, Romania; 4 2nd Internal Medicine Department, “Grigore T. Popa” University of Medicine and Pharmacy, 700115 Iasi, Romania

**Keywords:** GLP-1 receptor agonists, GIP, glucagon receptor agonist, dual agonists, triple agonists, neurodegeneration, neuroinflammation, neuroprotection

## Abstract

Glucagon-like peptide-1 receptor agonism has attracted interest in neurodegeneration because of its effects on metabolic and inflammatory pathways implicated in neuronal dysfunction. This narrative review examines the molecular rationale and neurological evidence for selective glucagon-like peptide-1 receptor agonists, dual agonists additionally targeting the glucose-dependent insulinotropic polypeptide receptor, and triple agonists also engaging the glucagon receptor. English-language literature retrieved from PubMed, Scopus, and Web of Science was synthesized according to study design and neurological relevance. Preclinical findings indicate modulation of insulin signaling, synaptic function, mitochondrial homeostasis, and neuroinflammation, but responses vary across compounds and experimental models. In Alzheimer’s disease, liraglutide did not meet its primary trial endpoint, and semaglutide did not slow clinical progression. Parkinson’s disease trials have yielded mixed results without establishing disease modification. Observational associations with reduced dementia risk remain susceptible to confounding. Neurological evidence for tirzepatide and retatrutide remains predominantly observational or preclinical. Greater metabolic efficacy does not establish neuroprotection, while nutritional vulnerability may constrain therapeutic applicability. Dedicated randomized trials integrating clinical outcomes with pharmacological exposure, target engagement, and metabolic mediation are required to distinguish systemic effects from direct neural actions and determine whether these therapies can prevent cognitive decline or modify established neurodegenerative disease.

## 1. Background: The Metabolic—Neurodegenerative Interface

The global rise in obesity is occurring in parallel with the increasing burden of disorders affecting the nervous system, creating an expanding epidemiological and clinical overlap between metabolic dysfunction and neurodegeneration [1,2]. Primary neurodegenerative diseases—including Alzheimer’s disease (AD), Parkinson’s disease (PD), multiple system atrophy (MSA), amyotrophic lateral sclerosis (ALS), and Huntington’s disease (HD)—together with multiple sclerosis (MS), a chronic immune-mediated disorder with a substantial neurodegenerative component, are increasingly investigated within a broader metabolic and vascular framework [3,4]. Obesity, type 2 diabetes mellitus (T2DM), metabolic dysfunction-associated steatotic liver disease, hypertension, and atherosclerotic vascular disease may influence neurological risk through insulin resistance, chronic inflammation, endothelial dysfunction, oxidative stress, and impaired metabolic flexibility [5,6,7]. The 2024 Lancet Commission recognized both obesity and diabetes as modifiable dementia risk factors, while emphasizing that risk-factor control is a prevention strategy rather than evidence of reversal of established neurodegeneration [7].

The obesity–neurodegeneration interface is supported by shared biological pathways, including impaired insulin signaling, mitochondrial dysfunction, chronic innate-immune activation, blood–brain barrier (BBB) and neurovascular-unit injury, altered lipid handling, defective autophagy, and disruption of neuronal energy homeostasis [3,6]. Brain insulin resistance can also influence peripheral metabolism and body-fat distribution, making the relationship bidirectional [8]. In AD, the phrase “type 3 diabetes” captures part of this biology, but should not be interpreted as implying that AD is simply a metabolic disorder or that glucose-lowering therapy will necessarily modify its core pathology [9].

These shared metabolic and inflammatory mechanisms have prompted interest in GLP-1-based therapies as potential interventions for neurological disease, building on their established efficacy in obesity and T2DM [10,11,12,13,14].

Endogenous glucagon-like peptide-1 (GLP-1) and glucose-dependent insulinotropic polypeptide (GIP) regulate glucose-dependent insulin secretion and exert broader effects on glucagon release, gastric emptying, appetite, adipose biology, and energy balance [13]. Selective GLP-1 receptor agonists (GLP-1RAs) are established therapies for T2DM and obesity [11,15,16]. Tirzepatide extends this strategy by combining GLP-1 receptor (GLP-1R) and GIP receptor (GIPR) agonism, while retatrutide and related molecules add glucagon receptor (GCGR) agonism. Because glucagon is not itself an incretin hormone, these compounds are more accurately described as GLP-1/GIP/glucagon receptor triple agonists (also termed triagonists) rather than “triple incretins” [11,13].

The metabolic efficacy of these agents creates an attractive but potentially misleading inference: if obesity and diabetes increase neurological risk, then greater reductions in body weight and glycated hemoglobin should translate into greater neuroprotection [15,17,18].

This may be plausible in prevention through improved vascular health, insulin sensitivity, and reduced systemic inflammation, but is less certain once established neurodegenerative pathology is present [12,19,20,21]. A molecule can be metabolically potent yet have limited access to relevant brain regions, insufficient target engagement, or no effect on the dominant disease pathway [18,22,23].

Three evidence streams must therefore be distinguished: mechanistic and preclinical studies testing biological plausibility; prevention-oriented human studies, separating randomized cardiometabolic trials from observational cohorts; and dedicated neurological randomized controlled trials (RCTs) evaluating clinical progression [18,20,21,24]. For claims of disease modification, dedicated neurological RCTs provide the most direct evidence. Neurological outcomes from cardiometabolic trials require assessment of prespecification and ascertainment; observational associations remain hypothesis-generating [18,20,21]. Mechanistic and animal findings alone cannot establish clinical efficacy. These categories are applied explicitly in Section 4 to distinguish biological plausibility, risk modification, and clinically meaningful disease modification.

This narrative review critically evaluates selective GLP-1RAs, dual GLP-1/GIP agonists, and emerging GLP-1/GIP/glucagon receptor triple agonists across AD, PD, MSA, MS, ALS, and HD. Particular emphasis is placed on receptor topography, central exposure, the hierarchy of preclinical, observational, biomarker, and randomized clinical evidence, and the distinction between risk reduction and clinically meaningful disease modification.

## 2. Literature Search and Selection

This narrative review was based on searches of PubMed/MEDLINE, Scopus, and Web of Science, last conducted on 1 September 2026. Searches focused on English-language publications published between 17 August 2021 and 17 August 2026, supplemented by earlier foundational studies identified through reference lists. Search terms combined “GLP-1”, “glucagon-like peptide-1”, “GIP”, “glucagon receptor agonist*”, and individual drug names with terms relating to neurodegeneration, Alzheimer’s disease, Parkinson’s disease, multiple system atrophy, multiple sclerosis, amyotrophic lateral sclerosis, Huntington’s disease, cognition, dementia, neuroinflammation, and neuroprotection. Detailed database-specific search strategies are provided in the Supplementary Material: PubMed/MEDLINE (Table S1), Scopus (Table S2), and Web of Science (Table S3).

Eligible literature included peer-reviewed clinical trials, observational studies, human mechanistic and pharmacokinetic investigations, preclinical studies, and relevant reviews addressing neurological outcomes, receptor signaling, CNS exposure, or treatment safety. Three authors reviewed titles, abstracts, and full texts, with final selection determined through discussion. Zotero was used for reference management. Earlier studies were retained when they provided foundational mechanistic evidence or informed the development of neurological trials. Publications outside the review’s scope, preprints, and conference abstracts were excluded.

Findings were synthesized thematically, with greatest weight assigned to dedicated neurological RCTs, followed by neurological outcomes from cardiometabolic RCTs, observational human studies, human mechanistic investigations, and preclinical evidence. The latter two categories informed biological plausibility, CNS exposure, and target engagement rather than clinical efficacy, particularly for dual and triple agonists. Systematic reviews and meta-analyses were interpreted according to their constituent studies. Regulatory communications and editorial notices contextualized adverse events and trial findings, while ongoing trials and published protocols outlined future research directions but were not treated as evidence of efficacy.

Favorable and unfavorable findings were interpreted using the same criteria, where applicable, including endpoint prespecification, multiplicity, outcome ascertainment, confounding, and concordance between biomarkers and clinical outcomes. No formal risk-of-bias assessment or certainty-of-evidence grading was performed.

## 3. The Obesity–Neurodegeneration Interface: Receptor Biology and Molecular Mechanisms of Selective, Dual, and Triple Agonism

### 3.1. Receptor Topography and Central Access

GLP-1R is a class B G protein-coupled receptor with canonical Gαs–adenylyl cyclase–cAMP signaling and context-dependent recruitment of additional pathways [11]. Brain mapping identifies dense receptor populations in circumventricular organs and brainstem or hypothalamic nuclei that sense circulating metabolic signals, including the area postrema, median eminence, arcuate nucleus, and nucleus tractus solitarius. Additional receptor-positive profiles have been described in limbic, thalamic, mesolimbic, and selected cortical or cerebellar territories. Ultrastructural localization on somata, dendrites, varicosities, and terminals supports both postsynaptic and presynaptic actions [25].

Importantly, central GLP-1 signaling is not solely dependent on gut-derived GLP-1. A distinct population of preproglucagon (PPG) neurons, located predominantly in the nucleus tractus solitarius, provides an endogenous source of GLP-1 within the CNS and projects to multiple GLP-1R-expressing regions involved in energy homeostasis, autonomic regulation, stress responses, and behavior [16]. This distinction is pharmacologically relevant, as activation of these endogenous circuits does not imply comparable access or target engagement by peripherally administered GLP-1RAs [16].

Central access should not be equated with BBB penetration. Experimental evidence indicates molecule- and method-dependent differences in the routes and extent of brain access among GLP-1RAs [22]. Fluorescently labeled semaglutide reached selected circumventricular and adjacent sites in rodents without broadly diffusing throughout protected brain parenchyma [23]. Comparable studies with GLP-1R/GIPR dual-agonist probes identified receptor-expressing cells in the pancreas and selected brain regions, but did not demonstrate unrestricted penetration beyond the pattern seen with single agonists [26]. Accordingly, plasma exposure, weight loss, or molecular size cannot be considered sufficient proxies for neuronal target engagement in the hippocampus, cortex, or substantia nigra [18,22,23,26].

GIPR is expressed in peripheral tissues and several brain regions [27], including hypothalamic nuclei involved in energy balance—the arcuate, dorsomedial, and paraventricular nuclei—as well as the area postrema and nucleus tractus solitarius [28]. GIPR expression has also been reported in oligodendrocytes and cellular components of the neurovascular unit, suggesting potential actions beyond classical neuronal metabolic circuits [28]. Experimental studies in mice support a functional role for central GIPR signaling: activation of GIPR-expressing GABAergic neurons reduces food intake and body weight, whereas disruption of neuronal GIPR signaling attenuates the metabolic effects of combined GIPR/GLP-1R agonism [29]. These findings support the involvement of central GIPR pathways in metabolic regulation but provide no direct evidence of therapeutic efficacy in human neurodegenerative disease.

Compared with GLP-1R and GIPR, central GCGR signaling is less well characterized. Studies nevertheless support a liver–brain axis downstream of peripheral GCGR activation [30,31]. Hepatic GCGR agonism can increase circulating FGF21, with central FGF21–β-Klotho signaling contributing to energy expenditure and weight loss [31]. GCGR agonism can also recruit GABAergic signaling within the mediobasal hypothalamus, promoting thermogenesis through a liver–brain–adipose axis [30]. The glucagon component of triple agonists may therefore engage central metabolic circuits indirectly, although extension of these mechanisms to neurodegenerative pathways requires disease-specific evidence.

The neurovascular unit provides a plausible interface between direct and indirect mechanisms. Endothelial cells, pericytes, astrocytic end-feet, microglia, and neurons collectively regulate barrier integrity, cerebral blood flow, nutrient delivery, and inflammatory trafficking. GLP-1R signaling may influence multiple components of this unit, with potential effects on endothelial and glial function, neurovascular coupling, and brain energy metabolism [32]. Neurological effects may therefore involve accessible neural and neurovascular compartments without implying broad distribution of GLP-1RAs across protected brain tissue [22].

### 3.2. Direct Neural Mechanisms and Indirect Metabolic–Vascular Pathways

Direct neural hypotheses are supported by early work showing that GLP-1 and exendin-4 promote neurite outgrowth, facilitate nerve growth factor-mediated differentiation, and protect neurons from excitotoxic or apoptotic injury [33,34]. GLP-1R activation also modulates neuronal calcium signaling and synaptic transmission, mechanisms relevant to synaptic plasticity and neuronal function in neurodegenerative disorders [35,36,37]. While these experimental observations support the biological plausibility of direct neural effects, they do not define the human dose, CNS exposure, treatment window, or degree of receptor engagement required for disease modification.

Indirect pathways may be at least as important. Sustained weight reduction can improve insulin sensitivity, sleep-disordered breathing, mobility, hepatic steatosis, blood pressure, and systemic inflammatory tone. Better glycemic control may reduce glucotoxicity and microvascular injury [19], while improved cardiovascular health may contribute to a lower cerebrovascular burden, including clinically overt and silent ischemic injury [38]. Semaglutide has demonstrated cardiovascular benefit in people with overweight or obesity without diabetes [12] and histological benefit in metabolic dysfunction-associated steatohepatitis [39]. Such systemic effects may influence neurological outcomes through improved vascular health, insulin sensitivity, and inflammatory status, independently of direct neuronal receptor engagement [21].

The distinction matters for interpretation. A reduction in incident dementia after years of metabolic treatment may reflect prevention of vascular injury, delayed crossing of a clinical diagnostic threshold, or residual confounding rather than a direct disease-modifying effect [21]. Conversely, failure to slow progression in biomarker-confirmed AD does not negate cardiometabolic value. Thus, an agent may confer population-level benefits for brain health without necessarily exerting a disease-modifying effect on established neurodegenerative pathology [21,24].

### 3.3. Convergent Intracellular Pathways

GLP-1R, GIPR, and GCGR share signaling through Gαs-mediated activation of adenylyl cyclase and cAMP production, although downstream pathway engagement depends on the receptor and cellular context. In experimental neural models, GLP-1R activation engages cAMP–protein kinase A (PKA)–cAMP response element-binding protein (CREB), PI3K–Akt, and MAPK/ERK signaling, with potential effects on neuronal survival, synaptic plasticity, and neurotrophic responses [37,40]. Liraglutide reduced amyloid-related oxidative and inflammatory stress in mice with AD-like pathology [41] and improved cognitive performance through mechanisms involving AMPK and PI3K/Akt signaling in diabetic rats [42].

Anti-inflammatory actions provide another proposed mechanism of GLP-1R-mediated neuroprotection. Experimental studies indicate that GLP-1R signaling can suppress NF-κB activity, NLRP3 inflammasome activation, inducible nitric oxide synthase, and pro-inflammatory cytokine production, while modulating microglial responses [18,37,40]. Single-cell and transcriptomic analyses showed that semaglutide attenuated neuroinflammatory programs in microglia, endothelial cells, and pericytes and reduced peripheral neutrophil infiltration in an LPS-challenged mouse model [43]. Such effects may contribute to neurovascular protection [32], but differences in drug exposure, peripheral immune responses, and experimental disease models limit extrapolation to human neurodegeneration [18].

GLP-1R activation has also been associated with improved mitochondrial function and cellular energy metabolism, reduced oxidative stress, and modulation of autophagic and mitophagic pathways [37,40]. A systematic review of experimental AD and PD models reported changes in autophagy-related markers following GLP-1R agonism, although their clinical relevance remains uncertain [44]. In some preclinical models, these mechanisms have been accompanied by reduced amyloid-β and tau pathology, preservation of dopaminergic neurons, and attenuation of α-synuclein-related pathology [17,40,45].

However, these findings are not consistent across experimental settings. Semaglutide did not improve amyloid pathology, glial markers, or behavioral outcomes in 5XFAD and APP/PS1 mice; both semaglutide and tirzepatide also yielded negative findings in younger 5XFAD mice despite preserved metabolic responses [46]. Similarly, preventive treatment with either agent for two or four months did not improve learning, memory, amyloid deposition, or glial activation in 5XFAD mice [47]. These results show that metabolic responses can occur without measurable neurological benefit, even when treatment begins before overt pathology. Taken together, the preclinical evidence supports further mechanistic investigation but does not establish consistent neuroprotection or equivalent CNS target engagement and disease modification in humans [17,45,46,47].

**Figure 1:**
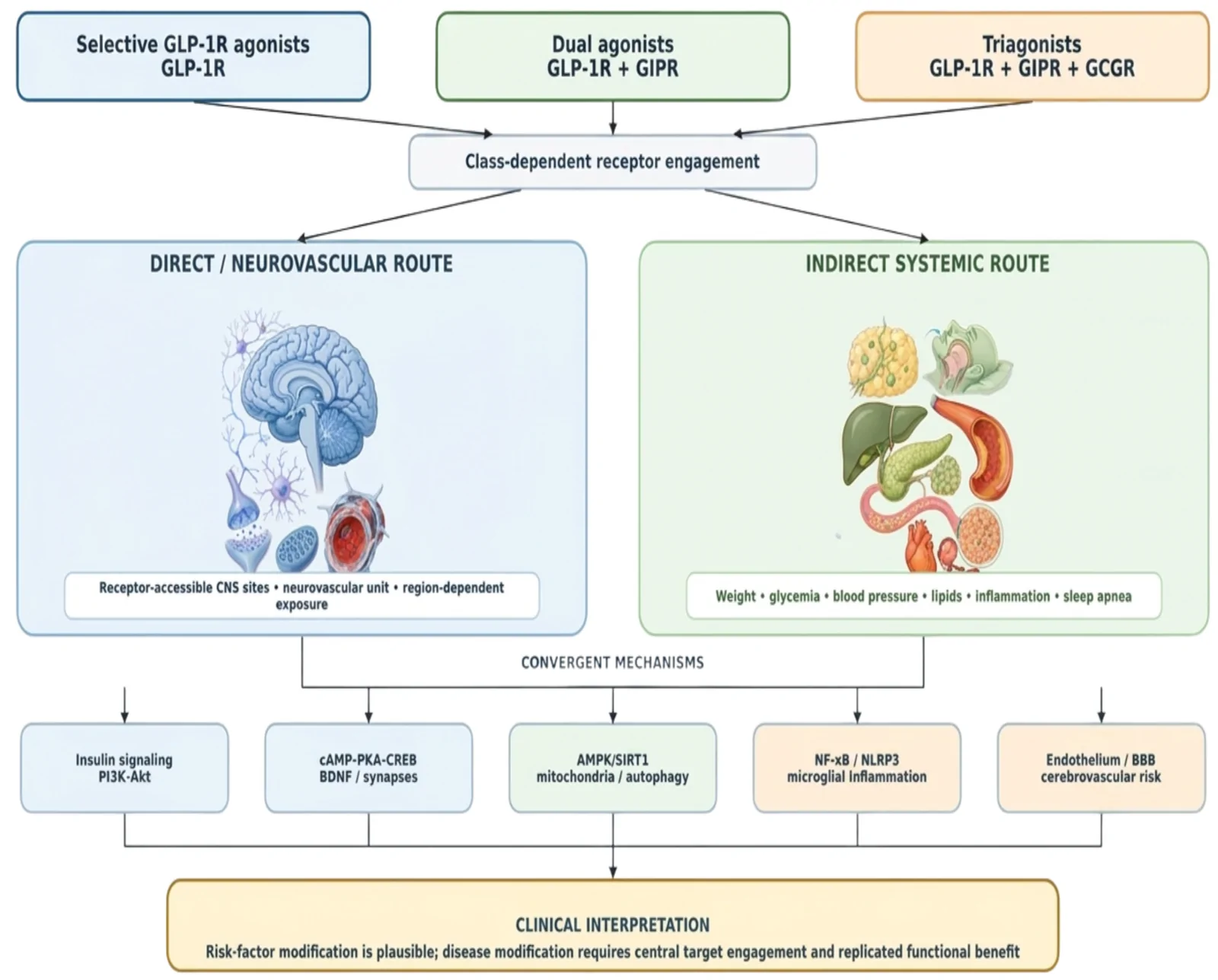
Metabolic Agonism and Neurological Outcomes: Direct, Indirect, and Convergent Pathways. Selective GLP-1R agonists, dual GLP-1R/GIPR agonists, and GLP-1R/GIPR/GCGR triple agonists may influence neurological outcomes through direct neurovascular and indirect systemic pathways converging on metabolic, mitochondrial, synaptic, inflammatory, and cerebrovascular mechanisms. These pathways illustrate biological plausibility but do not establish clinical disease modification. Created in BioRender. Popa, E. (2026) https://BioRender.com/9j0wnwg. Note: Triagonists denote GLP-1R/GIPR/GCGR triple agonists.

The proposed direct neurovascular and indirect systemic pathways, together with their convergent mechanisms, are summarized in Figure 1.

### 3.4. From Selective GLP-1R Agonism to Dual and Triple Agonism

Selective GLP-1R agonism provides the pharmacological foundation for dual- and triple-receptor approaches. Dual GLP-1R/GIPR agonism, exemplified by tirzepatide, combines GLP-1R-mediated actions with complementary GIPR signaling. The rationale extends beyond enhanced metabolic efficacy to potential modulation of neuronal and neuroimmune pathways, although these effects depend on receptor pharmacology and tissue exposure [48].

GLP-1R/GIPR/GCGR triple agonism incorporates GCGR-mediated effects on energy expenditure, lipid mobilization, and hepatic glucose metabolism alongside the anorectic and insulinotropic actions of incretin receptor activation [49,50]. These metabolic actions should be distinguished from the synaptic effects investigated with experimental triple- agonist peptides [49].

**Figure 2:**
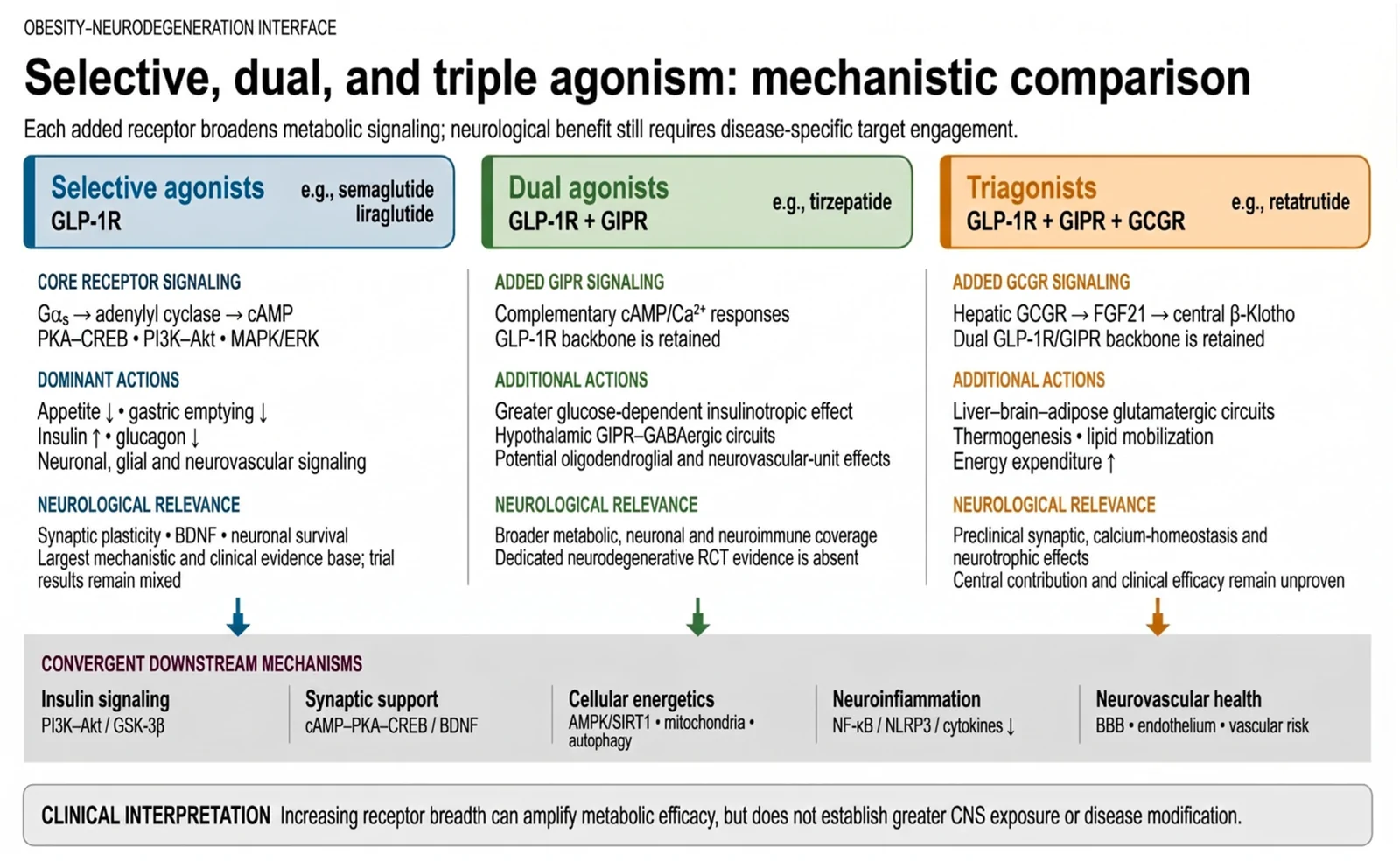
Mechanistic comparison of selective GLP-1R agonists, dual GLP-1R/GIPR agonists, and GLP-1R/GIPR/GCGR triple agonists at the obesity–neurodegeneration interface. Each additional receptor broadens metabolic and cellular signaling, while neurological relevance depends on disease-specific exposure and target engagement [18,48]. Convergent downstream mechanisms include insulin signaling, synaptic support, cellular energetics, neuroinflammatory modulation, and neurovascular effects [15,40,43]. Increasing receptor breadth may amplify metabolic efficacy but does not establish greater CNS exposure or disease modification [18,22,26]. Created in BioRender. Popa, E. (2026) https://BioRender.com/cg4vwx8. Note: Triagonists denote GLP-1R/GIPR/GCGR triple agonists.

The biological effects of multi-receptor agonists depend on the balance of activity at each receptor, tissue distribution, and CNS exposure. Increasing receptor breadth therefore does not, by itself, establish greater neural target engagement or neuroprotective activity [18,48]. Figure 2 integrates the receptor-specific and shared signaling pathways described above, distinguishing proposed neural effects from indirect metabolic and vascular mechanisms.

Table 1 places this mechanistic framework in the context of the available metabolic and neurological evidence and identifies the principal unresolved question for each pharmacological platform.

## 4. Comparative Therapeutic Evidence and Translational Perspectives Across Neurodegenerative Diseases

### 4.1. Alzheimer’s Disease and Cognitive Decline

Mechanistic and preclinical evidence supports the investigation of GLP-1-based therapies in AD. Brain insulin resistance, impaired cerebral energy metabolism, synaptic dysfunction, neuroinflammation, mitochondrial dysfunction, and vascular pathology provide multiple potential targets for GLP-1R-mediated effects [21,40,48]. Preclinical studies have reported reductions in amyloid burden, tau phosphorylation, oxidative stress, and cognitive impairment following GLP-1R agonism [17,40,48]. However, negative findings with semaglutide and tirzepatide in amyloid-based mouse models show that this preclinical rationale is not uniformly supported across agents and experimental settings [46,47].

Retatrutide-specific evidence relevant to cognitive outcomes remains preclinical. In streptozotocin-induced diabetic rats [64], treatment preserved overall Morris Water Maze performance relative to untreated diabetic animals and partially attenuated short-term passive avoidance deficits, without consistent normalization across memory measures. These findings were accompanied by a significant reduction in hippocampal TNF-α, a nonsignificant trend toward lower IL-1β, and partial preservation of cortical and hippocampal cytoarchitecture. Retatrutide alone did not improve behavioral performance beyond control levels. Direct central exposure was not established, and the relative contributions of systemic metabolic improvement and direct neural effects remain uncertain. Moreover, this insulin-deficient diabetes model does not reproduce AD-specific pathology, limiting extrapolation to Alzheimer’s disease [64].

Dedicated neurological RCTs have evaluated whether GLP-1R agonism translates into clinical benefit in established AD. In the ELAD study [52], 204 participants without diabetes and with mild-to-moderate AD were randomized to liraglutide or placebo for 52 weeks. The primary endpoint—change in cerebral glucose metabolism—was not significantly different between groups (estimated difference −0.17; 95% CI −0.39 to 0.06; *p* = 0.14). Exploratory secondary analyses suggested less decline in executive function and reduced gray-matter atrophy, but activities of daily living and global clinical outcomes did not show consistent benefit. Given the negative primary endpoint and the absence of multiplicity adjustment for secondary analyses, these signals were insufficient to confirm clinical efficacy [52].

Similarly, a meta-analysis of three randomized trials involving 278 participants with AD or MCI found no significant improvement in global cognition with liraglutide or exenatide compared with placebo (standardized mean difference −0.21; 95% CI −0.81 to 0.38). Although the small evidence base limits precision, these findings do not support a consistent cognitive benefit in the populations studied [65].

The EVOKE and EVOKE+ randomized trials [24] evaluated oral semaglutide in 3808 participants with early, amyloid-confirmed symptomatic AD. Neither trial demonstrated slower progression on the Clinical Dementia Rating–Sum of Boxes at 104 weeks: the estimated treatment difference was −0.08 (95% CI −0.35 to 0.20; *p* = 0.57) in EVOKE and 0.10 (95% CI −0.17 to 0.38; *p* = 0.46) in EVOKE+. Biomarker changes were not accompanied by clinical benefit, underscoring that such changes alone cannot establish disease modification [24].

A subsequent exploratory reanalysis of published aggregate EVOKE/EVOKE+ data identified a nominally significant functional difference at week 130 and trends in selected later outcomes [66]. However, these post hoc comparisons across outcomes and time points do not overturn the negative prespecified primary results or provide confirmatory evidence of disease modification.

Evidence from cardiometabolic RCTs provides a complementary perspective on potential cognitive risk reduction. A meta-analysis [20] reported lower odds of dementia or cognitive impairment among participants receiving GLP-1RAS (odds ratio 0.55; 95% CI 0.35–0.86). However, events were rare, and cognition was generally not a prespecified, systematically adjudicated outcome, limiting conclusions about neurological efficacy [20].

Observational studies have also reported favorable associations. In a propensity-matched electronic-health-record cohort of 60,860 adults with T2DM and obesity, semaglutide or tirzepatide use was associated with lower risks of dementia (hazard ratio 0.63; 95% CI 0.50–0.81), stroke, and mortality compared with the use of other antidiabetic medications; no significant association with PD was observed [59]. A separate large propensity-matched analysis associated GLP-1RA use with lower risks of selected neurodegenerative outcomes [67]. These findings remain hypothesis-generating because residual confounding and differences in prescribing, healthcare contact, and diagnostic ascertainment may influence the associations.

Within this observational evidence, a retrospective propensity-matched comparison [60] found lower risks of coded MCI (RR 0.12; 95% CI 0.06–0.22) and dementia (RR 0.15; 95% CI 0.09–0.26) among tirzepatide users than among semaglutide users, whereas the estimate for AD included the null (RR 0.48; 95% CI 0.22–1.01). Absolute MCI risks were low (0.025% versus 0.209%), and mean follow-up differed substantially (255 versus 507 days). Analyses accounting for time to event yielded a less pronounced association for MCI (HR 0.49; 95% CI 0.26–0.93) and nonsignificant associations for dementia and AD [60]. Healthy-adherer effects and treatment channeling by calendar period or access to care are additional potential explanations. Although follow-up began at treatment initiation, only diagnoses occurring at least 12 months later were counted; the handling of earlier events and censoring was insufficiently detailed to exclude selection or time-related bias. These concerns do not demonstrate that bias explains the findings, but they preclude interpreting RR 0.12 as an 88% treatment-induced reduction in MCI risk or evidence of superior neuroprotection [60].

Overall, dedicated AD trials have not demonstrated disease-modifying efficacy of GLP-1RAs [24,52]. Cardiometabolic trials and observational studies suggest a possible role in cognitive risk reduction, but do not establish prevention of AD or treatment of established disease [20,59,60,67]. The observational findings for tirzepatide and preclinical findings for retatrutide discussed here likewise do not demonstrate clinical disease modification or superior neuroprotection [60,64]. Dedicated neurological trials are needed to clarify their potential role in preventing cognitive decline or treating established AD.

### 4.2. Parkinson’s Disease and Related Synucleinopathies

Mechanistic and preclinical evidence supports the investigation of incretin-based therapies in PD, given the contribution of impaired insulin signaling, mitochondrial dysfunction, oxidative stress, neuroinflammation, and synaptic dysfunction to dopaminergic neurodegeneration [18,48]. Preclinical studies have reported neuroprotective effects of GLP-1R agonism in experimental models of PD, but translation into sustained clinical benefit has been inconsistent [18,40,48].

Dedicated neurological RCTs have tested this rationale in PD. In the LIXIPARK trial [54], 156 people with early PD received lixisenatide or placebo for 12 months. Motor disability progressed less in the active-treatment group, with a between-group difference of 3.08 points in MDS-UPDRS part III scores at 12 months (95% CI 0.86–5.30; *p* = 0.007), but gastrointestinal adverse events were common, and the durability and clinical relevance of the effect require confirmation [54]. Most secondary endpoints did not differ substantially between groups, and the post-washout analysis was not adjusted for multiplicity [54]. Thus, LIXIPARK identified a potential lixisenatide-specific therapeutic signal but did not establish a GLP-1RA class effect.

NLY01, a pegylated exenatide analogue developed for prolonged exposure, did not improve the combined Movement Disorder Society–Unified Parkinson’s Disease Rating Scale parts II and III at 36 weeks in 255 participants with early untreated PD [51]. Neither tested dose separated from placebo, and gastrointestinal adverse events were more frequent [51]. Earlier exenatide studies had suggested persistence of a motor benefit after washout [53], but the larger UK phase 3 trial involving 194 participants did not demonstrate benefit on primary or secondary clinical endpoints over 96 weeks [55].

Interpretation of the phase 3 exenatide findings requires additional caution. In June 2026, *The Lancet* issued an Expression of Concern regarding the phase 3 exenatide publication [56]. The trial report should therefore be considered alongside this notice; in the absence of sufficient publicly available information regarding its basis and implications, further speculation is unwarranted. Irrespective of this issue, the available clinical trial data do not currently demonstrate replicated efficacy of exenatide in PD.

In MSA, a randomized, open-label proof-of-concept trial involving 50 participants met its primary endpoint, showing less worsening of the Unified Multiple System Atrophy Rating Scale (UMSARS) parts I  +  II combined score at 48 weeks with exenatide than in controls. However, objective clinical measures, sensor-derived gait assessments, imaging, and biomarker findings did not show concordant benefits. Given the open-label design, expectation and observer bias may have contributed to the primary outcome difference. These findings therefore do not establish disease modification [68].

From a translational perspective, these findings highlight the need for future PD and MSA trials to characterize CNS exposure and target engagement, identify potentially responsive metabolic or inflammatory phenotypes, and distinguish symptomatic effects from genuine slowing of neurodegeneration [18,48]. The ongoing MOST-ABLE study, a phase 2 randomized, double-blind, placebo-controlled trial evaluating oral semaglutide in PD, reflects continued clinical investigation of this therapeutic strategy but, as a trial protocol, cannot yet inform efficacy [69].

### 4.3. Multiple Sclerosis, Amyotrophic Lateral Sclerosis, and Huntington’s Disease

Evidence in MS, ALS, and HD is considerably less mature than that available for AD and PD and remains predominantly preclinical [10,70,71,72]. In experimental autoimmune encephalomyelitis, GLP-1R activation reduced neuroinflammatory responses [73]. Liraglutide engaged AMPK/SIRT1 pathways and suppressed NLRP3-associated inflammation [74], while dulaglutide modulated pathogenic Th1/Th17 responses [75]. These mechanisms intersect with metabolic comorbidity and immune dysregulation in MS, but randomized trials evaluating neurological disability, relapse activity, or imaging outcomes have not yet been reported [71]. Accordingly, the present therapeutic relevance of GLP-1R agonism in MS relates primarily to treatment of coexisting metabolic disease and to the development of appropriately designed neurological trials, rather than to established disease modification [10,71].

ALS presents a distinct translational and safety challenge. Weight loss, hypermetabolism, and low body mass are associated with adverse prognosis, so the anorectic and weight-reducing effects that are advantageous in obesity may be undesirable or potentially harmful in metabolically vulnerable patients [70]. Reviews of IGF-1/GLP-1 signaling identify plausible anti-inflammatory, mitochondrial, and trophic mechanisms, but direct therapeutic data in human ALS remain insufficient [72]. Future studies of GLP-1-based therapies should therefore incorporate nutritional status, energy expenditure, body composition, and weight trajectory as major safety and stratification variables rather than secondary metabolic outcomes [70,76].

In HD models, liraglutide or exendin-4 improved neuronal insulin signaling, cognitive or motor phenotypes, pancreatic pathology, and survival [77,78,79]. Liraglutide also attenuated mutant huntingtin-associated neurotoxicity by restoring neuronal insulin signaling, linking impaired insulin regulation more directly to HD pathology [77]. These findings reinforce the relevance of insulin signaling and mitochondrial stress, but translational limitations are considerable: HD is genetically determined, unintended weight loss becomes increasingly prominent during disease progression [80] and no randomized trial has shown that GLP-1-based therapy modifies neurological progression [10].

MS, ALS, and HD therefore illustrate why the neurological effects of incretin-based therapies cannot be extrapolated uniformly across disorders [10]. An agent that is favorable in obesity-associated cognitive risk may be inappropriate in a catabolic motor-neuron disorder [70,72]. Likewise, an anti-inflammatory signal in an MS model may not alter compartmentalized inflammation in progressive MS [71], and restoration of insulin signaling in a toxin or transgenic model may not translate into a meaningful human endpoint [77,78,79]. Disease-specific biology, metabolic phenotype, and nutritional vulnerability should guide candidate selection and trial design rather than metabolic potency alone [10,70,71].

### 4.4. Dual and Triple Agonists: Molecular Rationale and Neurological Translation

Dedicated trials of selective GLP-1R agonists in AD and PD have yielded mixed or negative clinical results, without establishing a reproducible class-wide disease-modifying effect [24,51,52,53,54,55]. This experience provides a benchmark for evaluating the neurological relevance of dual and triple agonism.

For tirzepatide, observational human data support further investigation but do not demonstrate efficacy in neurodegenerative disease [59,60]. Comparisons with semaglutide remain susceptible to channeling bias, differences in baseline characteristics and access to care, and unequal follow-up [60]. Whether GIPR co-activation confers additional neurological benefit requires evaluation in dedicated randomized trials.

For triple agonists, interpretation requires a clear distinction between experimental peptides and retatrutide [50,61,62]. An experimental GLP-1R/GIPR/GCGR triagonist improved memory and measures of synaptic function, neuronal excitability, and calcium homeostasis in 3xTg-AD mice [49]. Cellular studies also demonstrated neurotrophic, neuroprotective, and anti-inflammatory effects of dual and triple agonists, with some responses exceeding those observed with exendin-4 under the tested conditions [63]. Retatrutide-specific evidence derives from streptozotocin-induced diabetic rats, in which treatment preserved spatial learning performance and partially attenuated behavioral and histological abnormalities, although direct CNS exposure was not demonstrated [64]. Human trials have demonstrated improvements in glycemic control and body weight but were not designed to evaluate disease modification in neurodegenerative disorders [50,61,62].

The principal translational question is whether broader receptor engagement can modify disease progression beyond systemic metabolic improvement. Addressing this question requires characterization of CNS exposure where relevant, evidence of receptor or pathway engagement, and concordance between mechanistic biomarkers and clinically meaningful outcomes [48,53]. The relative efficacy of selective, dual, and triple agonists in neurodegenerative disorders remains undetermined.

Table 2 summarizes the principal human studies relevant to the neurological translation of these pharmacological platforms.

## 5. Discussion: From Metabolic Efficacy to Neurodegenerative Disease Modification

Across selective GLP-1R agonists, dual GLP-1R/GIPR agonists, and GLP-1R/GIPR/GCGR triple agonists, a central translational question emerges: to what extent do metabolic efficacy and weight reduction predict neurological benefit? Current evidence suggests that these outcomes should be considered related but biologically distinct. Improvements in body weight, glycemic control, systemic inflammation, and cardiovascular risk may reduce upstream determinants of neurological disease, but they do not establish target engagement within disease-relevant neural or neurovascular compartments or modification of established neurodegenerative pathology [7,18,24].

### 5.1. What the Current Evidence Supports

Weight loss may contribute to brain health by reducing multiple upstream metabolic and vascular risk factors, including insulin resistance, hypertension, dyslipidemia, sleep apnea, systemic inflammation, hepatic metabolic stress, and cerebrovascular risk. Such effects may contribute to lower long-term dementia risk, particularly when obesity, diabetes, and vascular disease coexist [7]. The cardiovascular outcome evidence for semaglutide and the observational dementia and stroke signals are consistent with this prevention model [12,20,59].

The evidence does not support using weight change as a surrogate endpoint for modification of established neurodegeneration. EVOKE and EVOKE+ showed that substantial metabolic efficacy and changes in several biomarkers can occur without slowing clinical progression in early symptomatic AD [24]. PD trials similarly show that belonging to the GLP-1RA class does not predict replicated clinical benefit [51,54,55]. Metabolic improvement and neuroprotection may therefore coexist in some populations, but they should be regarded as biologically and clinically distinct outcomes.

A two-pathway model is therefore most appropriate [18,48]. The indirect pathway runs from metabolic treatment to weight and glycemic improvement, lower inflammatory and vascular burden, and reduced probability of future cognitive or cerebrovascular events. The direct pathway requires drug access to relevant neural or neurovascular targets, engagement of disease-linked signaling, and sustained effects on neurodegenerative biology. The pathways can coexist, but evidence supporting one should not be assumed to establish the other.

### 5.2. Weight Loss, Nutritional Vulnerability, and Patient Selection

The marked weight-reducing efficacy of GLP-1-based therapies requires careful patient selection in neurodegenerative disorders characterized by nutritional vulnerability, where further weight loss may represent a clinical risk rather than a therapeutic objective [12,76,81].

Unintentional weight loss is a clinically meaningful marker of nutritional vulnerability, functional decline, and adverse outcomes, although its relevance varies across disorders and disease stage [76,80,81]. In PD and HD, it may reflect dysphagia, gastrointestinal dysfunction, altered energy expenditure, motor impairment, behavioral changes, or disturbances of hypothalamic and reward-related pathways [80,81].

Nutritional status should therefore be assessed before intentional weight reduction is pursued, particularly in patients with established or anticipated nutritional vulnerability [76,82]. According to the Global Leadership Initiative on Malnutrition (GLIM), the diagnosis of malnutrition requires at least one phenotypic criterion—unintentional weight loss, low body mass index, or reduced muscle mass—and one etiologic criterion, comprising reduced food intake or assimilation or inflammation/disease burden [82].

Accordingly, the decision to use a GLP-1RA, a dual GLP-1R/GIPR agonist, or—within a trial—an investigational triple agonist should consider both the metabolic indication and the nutritional phenotype [10,76]. In patients with neurodegeneration who also have obesity, T2DM, or high cardiovascular risk, an approved agent may remain appropriate for its established metabolic indication when the expected cardiometabolic benefit outweighs the nutritional risk [10,12]. Conversely, further weight reduction may be undesirable in patients with low or rapidly declining body weight, sarcopenia, frailty, dysphagia, hypermetabolism, or an advanced catabolic phenotype, particularly in ALS and in later stages of HD or PD [76,80,81].

When treatment is justified by an established metabolic indication, weight trajectory should be interpreted alongside nutritional and functional status rather than as an isolated marker of therapeutic success [76,81]. Serial assessment of nutritional intake, swallowing and gastrointestinal symptoms, and, where feasible, muscle mass and strength may help identify increasing vulnerability [76,82]. Persistent unintended weight loss, worsening sarcopenia, or functional decline should prompt reassessment of the therapeutic strategy [76].

Preclinical evidence suggests that the neurological response to weight loss may differ from its metabolic effects. In an experimental model of diet-induced obesity, rapid dietary weight loss normalized glucose intolerance without reversing obesity-associated hypothalamic inflammation and was accompanied by further microglial and transcriptomic alterations [83]. Although these findings cannot be directly extrapolated to humans or incretin-based therapies, they illustrate that peripheral metabolic improvement and central neuroinflammatory resolution may follow different trajectories.

The clinical implications of weight reduction in neurodegenerative disorders should be evaluated in relation to baseline adiposity, nutritional status, and disease stage. Therapeutic goals should integrate cardiometabolic risk reduction with preservation of neurological function, maintenance of muscle mass, and patient-reported quality of life [7,12,76,80,81].

### 5.3. Translational Limitations and Safety Considerations

The timing of intervention may influence whether metabolic benefits translate into meaningful neurological outcomes. Addressing modifiable metabolic and vascular risk factors, particularly from midlife, offers a rationale for prevention before substantial neurodegenerative pathology develops [7]. Whether GLP-1 receptor agonists, dual agonists, or triple agonists provide additional protection against cognitive decline remains uncertain and requires dedicated prevention trials.

In established neurodegenerative disease, limited neurological efficacy may reflect late intervention, advanced neurodegeneration, or insufficient CNS exposure [18,84]. Species-related differences in GLP-1 receptor distribution may further complicate extrapolation from experimental models [85], while gastrointestinal adverse effects can limit treatment tolerability, as observed in LIXIPARK [54].

Endpoint choice is equally important. Motor scales are sensitive to medication state and symptomatic fluctuation; cognitive composites can be influenced by practice effects, dropout, and comorbidity; MRI volume may reflect fluid or inflammatory changes; and plasma biomarkers may respond to renal, hepatic, or weight-related physiology. Neurological trials should therefore seek convergence across prespecified clinical, imaging, fluid, and, where appropriate, digital outcomes [86]. Adequate multiplicity control and transparent handling of missing data should also be incorporated into trial design and interpretation [87].

The safety profile of these therapies should be interpreted in the context of disease-specific vulnerabilities. Nausea, vomiting, dehydration, gallbladder disease, and loss of lean mass may have greater consequences in older adults with frailty, dysphagia, autonomic failure, or low baseline weight [82]. Nutritional and catabolic vulnerability is particularly relevant in ALS and later-stage PD or HD [76,80,81]. The greater weight-reducing efficacy of dual GLP-1R/GIPR agonists and emerging GLP-1R/GIPR/GCGR triple agonists further emphasizes the importance of monitoring nutritional status and body composition in susceptible neurological populations [58,62].

Potential ocular adverse effects also warrant consideration. Observational studies have raised concerns about a possible association between GLP-1RA use, particularly semaglutide, and non-arteritic anterior ischemic optic neuropathy (NAION) [88,89]. The joint NANOS–AAO consensus statement highlights inconsistent findings, low absolute risk, and limitations related to confounding and diagnostic ascertainment [90]. The available evidence supports individualized benefit–risk assessment and systematic collection of ocular adverse events in neurological trials, but does not establish a uniform risk across selective, dual, and triple agonists.

The neuropsychiatric profile of GLP-1-based therapies is particularly relevant to their evaluation in neurological populations. Regulatory assessments have not identified evidence supporting a causal association between the therapies evaluated and suicidal ideation or behavior [91,92]. In a propensity-score matched cohort of patients with T2DM, semaglutide was not associated with higher 12-month risks of neurological or psychiatric outcomes, including suicidality, compared with other antidiabetic medications [93]. Evidence from randomized trials is similarly reassuring: a meta-analysis found no significant increase in suicide or self-harm events with GLP-1R agonists compared with placebo [94], while a post hoc analysis of the STEP 1, 2, 3, and 5 trials found no increased risk of depressive symptoms or suicidal ideation or behavior with semaglutide in participants without known major psychopathology [95]. Nevertheless, residual confounding in observational studies, limited follow-up, rare events, and selected trial populations constrain interpretation [93,94,95]. Generalizability to patients with neurodegenerative disorders remains uncertain, underscoring the need for dedicated trials with comprehensive neuropsychiatric assessment and longer-term safety monitoring.

### 5.4. A Biomarker- and Exposure-Informed Trial Roadmap

Future trials should enroll biologically characterized populations and define whether the therapeutic objective is primary prevention, secondary prevention, symptomatic benefit, or modification of established disease [18]. Metabolic stratification should include diabetes status, insulin resistance, adiposity, vascular burden, recent weight trajectory, and sarcopenia [7,76,81,82]. In populations vulnerable to nutritional decline, phenotyping should extend beyond body weight to include dietary intake, body composition, and measures of muscle mass or function, with disease-specific assessment of swallowing or autonomic dysfunction where clinically relevant [7,76,81,82]. Disease characterization should incorporate established biomarkers where available, including amyloid and tau measures in AD, dopaminergic imaging and α-synuclein-related biomarkers in PD, neurofilament light as a marker of neuroaxonal injury, and disease-appropriate imaging or fluid biomarkers in MS, MSA, ALS, and HD [96,97,98].

Pharmacological exposure should be measured rather than presumed. Trials should incorporate population pharmacokinetics, cerebrospinal-fluid or other disease-relevant compartment exposure where ethically and analytically feasible, receptor- or pathway-engagement markers, and dose–response analyses separated from weight loss [18,48].

Mediation analyses may then help determine the extent to which neurological effects are explained by changes in body weight, glycemia, blood pressure, systemic inflammation, or vascular events, and whether an effect remains compatible with direct neural or neurovascular mechanisms [99].

Treatment benefit should be assessed primarily through changes in cognition, motor function, disability, quality of life, and the time to disease progression. Biomarkers should complement these clinical outcomes, not replace them [18,86]. Multimodal assessment integrating clinical, imaging, fluid, and, where appropriate, digital biomarkers may strengthen disease characterization and treatment-response assessment [86]. Statistical methods, approaches to missing data, and any adjustments for multiplicity should be prespecified and transparently reported [87].

Beyond neurodegenerative diseases, future research may also examine these therapies in syndromes involving persistent neuroinflammation [100], metabolic dysregulation, and cognitive symptoms, including Long COVID [101]. This extension requires disease-specific mechanistic and clinical evidence, as shared features do not imply equivalent pathology or therapeutic response.

A credible claim of neuroprotection would require concordant effects on clinically relevant neurological outcomes and, where feasible, disease-relevant biomarkers of neuroinflammation, axonal injury, synaptic dysfunction, or neurovascular pathology, rather than improvement in body weight, glycemic control, or systemic inflammation alone [18,24]. Replicated benefit across independent, adequately powered trials would be required before selective GLP-1R agonists, dual GLP-1R/GIPR agonists, or GLP-1R/GIPR/GCGR triple agonists could be considered disease-modifying neurological therapies [18,21,87].

### 5.5. Methodological Limitations of This Review

This narrative review compared selective GLP-1R agonists, dual GLP-1R/GIPR agonists, and GLP-1R/GIPR/GCGR triple agonists across heterogeneous neurological disorders. Restriction to English-language publications, emphasis on recent literature, and selective inclusion of earlier studies may have excluded relevant evidence. Selection, citation, and publication bias remain possible despite inclusion of favorable and unfavorable findings. No formal risk-of-bias assessment or certainty-of-evidence grading was undertaken; evidence was weighted qualitatively according to study design, clinical relevance, and methodological limitations.

Comparisons are constrained by unequal evidence maturity: dedicated neurological RCTs mainly concern selective GLP-1R agonists, whereas evidence for tirzepatide and retatrutide is largely observational or preclinical. Differences in populations, models, exposure, endpoints, and follow-up limit comparative conclusions. Inadequate assessment of CNS exposure, target engagement, and metabolic mediation also limits separation of direct neural actions from systemic effects. The evidence categories used here improve transparency but do not eliminate these uncertainties. Observational associations cannot establish disease modification, and preclinical or biomarker findings cannot substitute for clinical benefit in dedicated neurological trials.

## 6. Conclusions

Selective GLP-1R agonists, dual GLP-1R/GIPR agonists, and GLP-1R/GIPR/GCGR triple agonists provide a pharmacological framework for investigating how metabolic modulation may influence neurodegenerative processes. Selective GLP-1R agonists have the most extensive neurological evidence base, but dedicated trials in AD and PD have yielded mixed or negative results, without demonstrating a reproducible class-wide disease-modifying effect. Neurological evidence for dual and triple agonists remains predominantly observational or preclinical and does not establish superiority over selective agents. Greater metabolic efficacy alone cannot substantiate claims of neuroprotection.

A central unresolved question is whether these therapies influence neurological outcomes through systemic metabolic and vascular improvements, direct neural actions, or both. Weight reduction is not a validated surrogate for neurological benefit, and its clinical implications depend on baseline adiposity, nutritional reserves, and disease stage. In patients with frailty, sarcopenia, or involuntary weight loss, preservation of nutritional status and neurological function deserves particular consideration.

Further progress requires disease-specific randomized trials integrating clinically meaningful outcomes with assessment of pharmacological exposure, target engagement, and metabolic mediation. Such studies should clarify which patients may benefit and whether treatment can prevent cognitive decline or modify established neurodegenerative disease.

## Figures and Tables

**Table 1:** Comparative pharmacological platforms and current neurological evidence.

Platform	Representative Agents	Established Metabolic Role	Neurological Evidence	Principal Unresolved Question
Selective GLP-1R agonism	Exenatide, liraglutide, lixisenatide, semaglutide; NLY01 (investigational pegylated exenatide analogue)	Approved GLP-1RAs are used for T2DM and/or obesity, with cardiovascular benefit demonstrated for selected agents [11,12].	Preclinical findings are heterogeneous, with both favorable and null results [40,46,47]. Dedicated AD and PD trials show mixed or negative clinical results [24,51,52,53,54,55,56].	Can GLP-1R agonism produce clinically meaningful neurological benefits beyond metabolic and vascular risk modification?
Dual GLP-1R/GIPR agonism	Tirzepatide	Tirzepatide has demonstrated substantial glycemic and weight-reducing efficacy in T2DM and obesity [57,58].	Observational cognitive and neurodegenerative associations [59,60] and mechanistic rationale [48] support further investigation; however, negative findings in amyloid-based mouse models indicate inconsistent preclinical efficacy [46,47]. Clinical neurological efficacy remains unproven.	Does GIPR co-activation add disease-relevant neural effects beyond systemic metabolic improvement?
GLP-1R/GIPR/GCGR triple agonism	Retatrutide and experimental peptides	Retatrutide has demonstrated metabolic efficacy in clinical proof-of-concept, phase 2, and phase 3 studies [50,61,62].	Experimental triple agonists show cognitive and synaptic effects in rodent models [49] and neurotrophic and anti-inflammatory effects in cellular models [63]. Retatrutide-specific findings derive from diabetic rats [64]; these findings do not demonstrate clinical neurological efficacy.	Does GCGR engagement contribute disease-relevant neural effects beyond systemic metabolic change and weight reduction?

Note: The phase 3 exenatide trial in Parkinson’s disease is subject to an Expression of Concern and should be interpreted alongside this editorial notice [56]. Abbreviations: AD, Alzheimer’s disease; GCGR, glucagon receptor; GIPR, glucose-dependent insulinotropic polypeptide receptor; GLP-1R, glucagon-like peptide-1 receptor; GLP-1RAs, GLP-1 receptor agonists; PD, Parkinson’s disease; T2DM, type 2 diabetes mellitus.

**Table 2:** Selected human evidence most relevant to neurological translation.

Agent and Study	Population/Design	Primary Neurological Result	Key Limitation	Interpretation
Liraglutide—ELAD [52]	204 participants, mild-to-moderate AD, 52-week phase 2b RCT	Primary cerebral glucose-metabolism endpoint not significant (*p* = 0.14); exploratory executive/MRI signals	Negative primary endpoint; secondary analyses exploratory	Mechanistic signal, not established clinical efficacy
Oral semaglutide—EVOKE/EVOKE+ [24]	3808 participants, early amyloid-confirmed AD, two 104-week phase 3 RCTs	No slowing of CDR-SB in either trial	Biomarker changes were not accompanied by clinical benefit	Strong evidence against disease modification in the tested AD population
Lixisenatide—LIXIPARK [54]	156 participants, early PD, 12-month phase 2 RCT	Modest attenuation of motor progression	Gastrointestinal adverse events; replication and durability needed	Promising molecule-specific signal
NLY01 [51]	255 participants, early untreated PD, 36-week phase 2 RCT	No benefit on MDS-UPDRS II + III at either dose	Relatively short duration; no validated target-engagement signal	Negative clinical efficacy trial
Exenatide—UK phase 3 [55,56]	194 participants with PD, 96-week phase 3 RCT	No primary or secondary clinical benefit reported	Lancet Expression of Concern issued June 2026	No replicated phase 3 efficacy; interpret report cautiously
Exenatide—MSA [68]	50 participants with MSA; randomized, open-label proof-of-concept trial.	UMSARS I + II endpoint met (48 weeks); no supporting objective, imaging, or biomarker benefit.	Open-label design and expectation bias	Insufficient evidence of disease modification
Semaglutide or tirzepatide—TriNetX [59]	60,860 matched adults with T2DM and obesity; retrospective cohort: semaglutide or tirzepatide versus other antidiabetic drugs.	Lower coded dementia, stroke, and mortality; no significant PD association	Residual confounding; treatment selection; outcome coding	Hypothesis-generating associations; no demonstrated causal neuroprotection or disease modification.
Tirzepatide vs. semaglutide [60]	44,470 matched participants per group; retrospective cohort	Lower MCI and dementia coding with tirzepatide; AD estimate imprecise	Low absolute risks, limited follow-up, channeling bias	Hypothesis-generating comparison; no demonstrated causal superiority of tirzepatide.
Retatrutide [62]	Phase 3 T2DM trial; no neurodegenerative-disease population	No neurological efficacy endpoint	Metabolic trial cannot establish central target engagement	Metabolic proof of concept only

Abbreviations: AD, Alzheimer’s disease; CDR-SB, Clinical Dementia Rating–Sum of Boxes; MCI, mild cognitive impairment; MDS-UPDRS, Movement Disorder Society–Unified Parkinson’s Disease Rating Scale; MSA, multiple system atrophy; PD, Parkinson’s disease; RCT, randomized controlled trial; T2DM, type 2 diabetes mellitus; UMSARS, Unified Multiple System Atrophy Rating Scale.

## Data Availability

No new data were created or analyzed in this study. Data sharing is not applicable to this article.

## Supplementary Materials

The following supporting information can be downloaded at: https://www.mdpi.com/article/10.3390/ijms27198738/s1.

## Abbreviations

The following abbreviations are used in this manuscript:

3xTg-AD	Triple-transgenic Alzheimer’s disease mouse model
5XFAD	Five familial Alzheimer’s disease mutations mouse model
AAO	American Academy of Ophthalmology
AD	Alzheimer’s disease
Akt	Protein kinase B
ALS	amyotrophic lateral sclerosis
AMPK	AMP-activated protein kinase
APP/PS1	Amyloid precursor protein/presenilin 1
BBB	blood–brain barrier
BDNF	brain-derived neurotrophic factor
cAMP	Cyclic adenosine monophosphat
CDR-SB	Clinical Dementia Rating–Sum of Boxes
CI	confidence interval
CNS	central nervous system
COVID-19	Coronavirus disease 2019
CREB	cAMP response element-binding protein
FGF21	fibroblast growth factor 21
GABA	Gamma-aminobutyric acid
Gαs	Stimulatory G protein alpha subunit
GCGR	glucagon receptor
GIP	glucose-dependent insulinotropic polypeptide
GIPR	glucose-dependent insulinotropic polypeptide receptor
GLIM	Global Leadership Initiative on Malnutrition
GLP-1	glucagon-like peptide-1
GLP-1R	glucagon-like peptide-1 receptor
GLP-1RA	glucagon-like peptide-1 receptor agonist
GSK-3β	glycogen synthase kinase-3 beta
HD	Huntington’s disease
HR	Hazard ratio
IGF-1	insulin-like growth factor 1
IL-1β	interleukin-1 beta
LPS	lipopolysaccharide
MAPK/ERK	mitogen-activated protein kinase/extracellular signal-regulated kinase
MCI	mild cognitive impairment
MDS-UPDRS	Movement Disorder Society–Unified Parkinson’s Disease Rating Scale
MRI	magnetic resonance imaging
MS	multiple sclerosis
MSA	multiple system atrophy
NAION	Non-arteritic anterior ischemic optic neuropath
NANOS	North American Neuro-Ophthalmology Society
NF-κB	nuclear factor kappa B
NLRP3	NLR family pyrin domain containing 3
OR	Odds ratio
PD	Parkinson’s disease
PI3K	phosphoinositide 3-kinase
PKA	protein kinase A
PPG	preproglucagon
RCT	randomized controlled trial
RR	Risk ratio
SIRT1	sirtuin 1
T2DM	type 2 diabetes mellitus
Th1/Th17	T helper type 1/type 17 cells
TNF-α	tumor necrosis factor alpha
UMSARS	Unified Multiple System Atrophy Rating Scale
