# Supplementary material for: GLP-1, GIP, and Glucagon Receptor Agonism at the Obesity–Neurodegeneration Interface: A Narrative Review
Source: Int J Mol Sci. 2026 Sep 30;27(19):8738. doi: 10.3390/ijms27198738 (PMC13664769; doi:10.3390/ijms27198738)
Supplement: Supplementary file 1 [file ijms-27-08738-s001.zip › Supplementary_Search_Strategies.pdf]

Supplementary Search Strategies

GLP-1, GIP, and Glucagon Receptor Agonism at the Obesity–Neurodegeneration Interface

Access to subscription-based databases and full-text publications was provided through the institutional electronic resources of “Grigore T. Popa” University of Medicine and Pharmacy, Iasi, Romania.

Table S1. PubMed/MEDLINE search strategies

**Database**	**PubMed/MEDLINE**
**Last search**	1 September 2026
**Scope and limits**	Title and abstract; English-language publications; publication window 17 August 2021–17 August 2026. Publication cutoff: 17 August 2026.
**Search string**	("GLP-1"[tiab] OR "glucagon-like peptide-1"[tiab] OR GIP[tiab] OR "glucagon receptor agonist*"[tiab] OR semaglutide[tiab] OR liraglutide[tiab] OR exenatide[tiab] OR lixisenatide[tiab] OR dulaglutide[tiab] OR tirzepatide[tiab] OR retatrutide[tiab] OR NLY01[tiab]) AND (neurodegenerat*[tiab] OR Alzheimer*[tiab] OR Parkinson*[tiab] OR "multiple system atrophy"[tiab] OR "multiple sclerosis"[tiab] OR "amyotrophic lateral sclerosis"[tiab] OR Huntington*[tiab] OR cognit*[tiab] OR dementia[tiab] OR neuroinflammat*[tiab] OR neuroprotect*[tiab]) AND english[lang] AND 2021/08/17:2026/08/17[dp]

Note. The publication cutoff is distinct from the date on which the searches were last conducted. Earlier foundational studies were identified through backward reference screening. Three authors reviewed titles, abstracts and full texts, with final study selection determined through discussion.

Table S2. Scopus search strategies

**Database**	**Scopus**
**Last search**	1 September 2026
**Scope and limits**	Title, abstract and keywords; English-language publications; publication years 2021–2026. Eligible publication window: 17 August 2021–17 August 2026.
**Search string**	TITLE-ABS-KEY(("GLP-1" OR "glucagon-like peptide-1" OR GIP OR "glucagon receptor agonist*" OR semaglutide OR liraglutide OR exenatide

	OR lixisenatide OR dulaglutide OR tirzepatide OR retatrutide OR NLY01) AND (neurodegenerat* OR Alzheimer* OR Parkinson* OR "multiple system atrophy" OR "multiple sclerosis" OR "amyotrophic lateral sclerosis" OR Huntington* OR cognit* OR dementia OR neuroinflammat* OR neuroprotect*)) AND PUBYEAR > 2020 AND PUBYEAR < 2027 AND LIMIT-TO(LANGUAGE, "English")

Note. PUBYEAR restricts retrieval to the full calendar years 2021–2026. Eligibility within the exact publication window (17 August 2021–17 August 2026) requires verification of individual publication dates during screening.

Table S3. Database-specific search strategies -Web of Science

**Database**	**Web of Science**
**Last search**	1 September 2026
**Scope and limits**	Topic; English-language publications; publication window 17 August 2021–17 August 2026. Publication cutoff: 17 August 2026.
**Search string**	TS=(("GLP-1" OR "glucagon-like peptide-1" OR GIP OR "glucagon receptor agonist*" OR semaglutide OR liraglutide OR exenatide OR lixisenatide OR dulaglutide OR tirzepatide OR retatrutide OR NLY01) AND (neurodegenerat* OR Alzheimer* OR Parkinson* OR "multiple system atrophy" OR "multiple sclerosis" OR "amyotrophic lateral sclerosis" OR Huntington* OR cognit* OR dementia OR neuroinflammat* OR neuroprotect*)) AND LA=(English) AND DOP=2021-08-17/2026-08-17

Note. English-language and publication-date restrictions are included in the search string. DOP specifies the inclusive interval 17 August 2021–17 August 2026; records with incomplete publication dates require verification during screening.
